# Age Friendly Characteristics and Sense of Community of an Italian City: The Case of Macerata

**DOI:** 10.3390/ijerph20105847

**Published:** 2023-05-17

**Authors:** Paola Monachesi

**Affiliations:** Department of Languages, Literature and Communication, Utrecht University, Trans 10, 3512 JK Utrecht, The Netherlands; p.monachesi@uu.nl

**Keywords:** age-friendly cities, sense of community, urban and rural ageing, case study, landscape, later-life migration, Marche region, Italy

## Abstract

The paper presents a study about the city of Macerata, as a representative case of an urban community in the Marche Region, Italy. The aim of this paper is to assess the level of its age-friendliness by means of a quantitative analysis based on a questionnaire that relies on the well-established eight AFC domains proposed by the WHO. In addition, the sense of community (SOC) is investigated and how the older residents relate to it. Studies that analyze age-friendly Italian cities in relation to elder outcomes are limited. The paper contributes to fill this gap, and the findings reveal that the elderly respondents are not particularly satisfied about the services and the urban infrastructure of the city but show nevertheless a sense of community. It might be the combination of urban and rural features that contributes to the longevity and strong sense of community of the city despite its poor infrastructure and average services.

## 1. Introduction

The creation of age-friendly environments to promote elderly health and well-being has received increasing interest from academic and public policy fronts.

The Age-friendly cities (AFC) initiative launched by the WHO was based on the active ageing framework. It aims at improving quality of life of the ageing population by optimizing opportunities with respect to health, participation, security, and quality of life [1,2]. Environments play an important role in determining health and well-being, as well as how individuals adjust to loss of function and other forms of adversity that one might experience in life, especially in later years [3].

At the basis of the AFC initiative there was a project examining the experiences of older people living in urban areas through large-scale focus groups sessions with various stakeholders. This research contributed to identify key features of an age-friendly community [4] with respect to services, building environments, and social aspects. The results were identified in a guide and a checklist in which these characteristics were grouped under eight domains of urban life. These tools have become an important instrument for cities to evaluate the level of their age-friendliness and possible ways to improve it [5]. In addition, the Global Network of Age-Friendly Cities and Communities (GNAFCC) was launched so that cities that decide to join it can collaborate and exchange experiences. Now, it includes 1445 cities and communities in 51 countries, including over 300 million people world-wide. However, only four Italian cities have joined the network.

As result of this initiative, empirical research in this area has flourished with a focus on the way cities and communities respond to the needs of the ageing population. To this end, surveys have been developed, based on the WHO AFC framework, to assess the perceived age-friendliness of individuals with respect to the urban context in which they live (cf. for example [6,7,8,9,10,11,12]).

While age-friendly characteristics of cities have been assessed in several studies, the subject of investigation has mainly concerned urban areas [13,14], especially in North America and in European countries [15,16,17,18,19,20]. Recently, various studies have focused on high density and high-rise cities in the Asia and Pacific region, such as Hong Kong [6,7,8,9,10,11]. Research on age-friendliness of rural communities is more limited, but initiatives followed a Canadian project launched in parallel to the WHO one. The initiative was mainly concerned with rural and remote communities, triggered by the fact that a third of Canadian adults above 65 years old live in rural areas [21]. The same WHO protocol was followed, and guidelines and features were identified to make rural communities more age-friendly [22]. Initiatives have followed in Australia [23], Canada [24], and Ireland [25].

Studies that analyze the age-friendliness of Italian cities are very limited. There has been little empirical research examining age-friendly environments in relation to elder outcomes in Italy despite its rapidly growing elderly population. However, these studies could be beneficial and crucial in developing appropriate policies given its ageing population. From a demographic perspective, Italy is the second oldest country in the world and the oldest in Europe [26].

This paper aims to fill the gap with an empirical study about the city of Macerata, as representative case study of an urban community in the Marche Region. Macerata is the county seat of its own province. The goal is to assess the level of its age-friendliness by means of a quantitative analysis based on a questionnaire that relies on the eight AFC domains of the WHO checklist. In addition, sense of community (SOC) is investigated and how the inhabitants relate to it.

A quantitative approach has been adopted to collect primary data on residents’ opinion towards the age-friendly characteristics of the city. It is relevant to assess how the ageing population perceives the available infrastructure and services, as well as the social activities organized and whether they reflect the needs of the elderly. It is also important to investigate whether they affect the sense of community that can play an important role in the ageing process. Therefore, the survey also includes an assessment of sense of community in relation to socio-demographic features and the AFC domains. Sense of community is a term used to characterize the relationship between the individual and the social structure usually related to community participation [27,28].

The survey has been carried out in Macerata for several reasons. It represents an interesting case study given the longevity that characterizes the region and its cities. It seems thus relevant to investigate whether longevity could be associated with age-friendly characteristics of the urban environment. Furthermore, the region is becoming a new frontier for international retirement migration because of its pleasant lifestyle and wonderful landscape [29]. In addition, the city presents both urban and rural features and constitutes thus a different setting from the global cities or isolated rural communities investigated in the context of AFC studies and thus worth special attention.

The survey at the basis of this study and the research related to it have been carried out in the context of the European project Grage (https://www.grageproject.eu/ (accessed on 31 December 2022)) that focused on the challenges of ageing and sustainable development in urban areas. The research has evolved around the idea of citizenship, healthy environment, and suitable urban solutions for an ageing society. The themes addressed in the project are elderly legal rights, green urbanization, food sustainability, and analysis of elderly urban behavior. The project was carried out in the period 2014–2018.

The paper is innovative in several respects. At the *theoretical level*, it contributes to the age-friendly cities literature by providing a relevant case study for Italy, a country that despite its growing ageing population has not been studied extensively from this perspective. While the age-friendliness of the cities based on the eight WHO domains has been mainly tested in the context of high-density, high-rise big cities, it seems necessary to also consider an urban setting which shares many of the city characteristics and infrastructure, but which is also characterized by a rural history and background. It becomes thus possible to assess whether the features identified through the WHO checklist are good indicators to investigate the age-friendliness of urban environments with rural roots such as Macerata.

At *the policy level*, there is the need to develop good policies that consider the ageing process, especially in Italy where the peak of ageing will hit in 2045–50, when almost 34% of the population will be 65 years and older [30]. It could be relevant to learn from the Marche experience, given the longevity of its inhabitants and the impact that infrastructure and services, as well as landscape and rural roots, might have in an urban context.

## 2. Materials and Methods

### 2.1. Location of the Study Area

This study is based on a survey conducted in the city of Macerata, in the Marche region (Italy), and it examines the perceived age-friendliness of its elderly population.

The Marche region is located on the eastern side of the Italian peninsula, facing the Adriatic Sea. It exhibits an ageing population due to a decline process both in terms of mortality as well as of fertility, a trend that it shares with other Italian regions [31]. As a consequence of this process, longevity characterizes the Marche region: the oldest living Italian aged 112 lives there, as well as a growing number of people aged 100 and above, with 9 of them having reached 107. The reasons for this longevity might be found in the good quality of life which makes it a desirable place to live also for elderly people coming from abroad. The Marche region is becoming a new frontier for international retirement migration, with a growing number of older people, especially from Northern Europe choosing to settle there. These later-life migrants are attracted by a lifestyle based on active ageing, care for the environment, quality of life, and the possibility they have of becoming part of a rather authentic rural-based society [29].

Macerata is a city of about 40k inhabitants, and it is located at the intersection of three physical zones that characterize the region: hills, mountains, and coastline. It is a medieval fortified city located on top of a hill and surrounded by a characteristic landscape made of rolling hills, valleys, and rivers. It is about 30 km from the Adriatic Sea and the coastline, where much of the region’s population is concentrated in settlements dedicated to industry, fishing, and tourism. The mountains with the Apennine chain are about 50 km away, in the region’s interior. Macerata is home to an old university and to many administrative offices and banks. The climate is typically Mediterranean, with hot dry summers and mild to cool rainy winters.

The city has an ageing population due to depopulation driven by a combination of outmigration and sub-replacement fertility [32]. These are processes shared by rural Italy, but in the Marche region, they are related to the ending of the mezzadria share-tenancy system. This is an agrarian regime that has forged the landscape surrounding Macerata, which is characterized by small fields, vineyards, orchards, pastures, and woodland as well as many hills dominated by ancient towns and numerous farmhouses and cottages [29].

It seems thus relevant to investigate whether this favorable context for ageing attested in the Marche region reflects in the perception of its inhabitants by evaluating the age friendliness of its urban communities, by focusing on the city of Macerata, as a case study.

### 2.2. Procedures and Participants

A quantitative approach is adopted to collect primary data on residents’ opinion towards the age-friendly characteristics of the city of Macerata.

The survey employed a convenience sampling method, and participants have been recruited from a variety of settings including elderly centers, community halls, summer vacation camps for the elderly, churches, university offices, and public offices. Geographical variation was considered so that participants were drawn from various parts of the city and consequently from different socio-economic profiles.

Data were collected by face-to-face interviews with the elderly while younger (i.e., between 55 and 67) and literate respondents administered their own questionnaire. Filling the questions would take between 45 and 60 min.

The respondents are individuals belonging to two age groups, that is between 55 and 64 (pre-retirement) and older than 64 (retired). Including ‘soon to be old’ individuals (i.e., between 55 and 64) besides the aged ones can be revealing since they often act as caregivers of their old parents and can provide valuable insights on how age-friendly cities should be designed. Collecting data from both these age groups allows for a more comprehensive view on needs and requirements of the elderly.

Participants are professionals and officers working in the municipality and at the university, as well as retired people. In total, 163 respondents filled out the questionnaire. Researchers helped a few aged participants who had difficulties in reading or writing by reading the questions and writing down their responses to the items.

As already mentioned, the data were collected in the context of the European project Grage, which focused on the challenges of ageing and sustainable development in urban areas. Therefore, research carried out, including this study, has met the privacy and ethics requirements stated in the project Grant Agreement. Participants were provided details about the procedure while informed consent was sought by the respondents prior to filling the questionnaire.

### 2.3. Socio-Demographic Variables

Socio-demographic variables collected in this study include age, sex, education level, marital status, type of housing, total length of time having lived in the neighborhood, living arrangement, economic activity status, occupation, subjective health, experience in taking care of elderly, experience in taking care of children, use of elderly centers, and income.

Subjective health, assessed by a single question ‘how would you rate your overall health at present time?’, was measured using five-point Likert-type scales, ranging from 1 (“bad”) to 5 (“excellent”) [10]. Financial satisfaction (e.g., monthly financial resources available to pay for fixed expenses) was measured using a five-point Likert scale from 1 (“very insufficient”) to 5 (“very sufficient”) [33,34]. With respect to the type of housing, the choice was between public (subsidized) rental housing, private rental housing, (subsidized) ownership housing, and retirement home. As for living arrangements, subjects were asked whether they live with a partner, with children, with a partner and children, or alone.

### 2.4. The Age-Friendly City Scale (AFC) and Sense of Community scale (SOC)

In this study, data were collected through a questionnaire developed by [6] for Hong Kong. It is based on an 85 item checklist of the essential features of age-friendly cities [35] and it includes 58 items, in addition to 16 demographic questions, previously discussed. Its aim is to assess the perception of old adults on whether the urban environment they live in is age-friendly by considering the well-established eight WHO AFC domains. They comprise outdoor spaces and buildings (9 items), transportation (12 items), housing (4 items), social participation (7 items), respect and social inclusion (7 items), civic participation (3 items), communication and information (10 items), community, and health services (7 items). The questionnaire has been employed and validated in several earlier studies [6,7,8,9,10].

The questionnaire has been translated into Italian and adapted to the Italian context; more specifically, three additional questions related to ICT and technology have been added given their relevance for the future of urban ageing (cf. also [12]).

The level of age-friendliness on each listed item was quantified on a 6-point Likert scale, ranging from 1 (‘strongly disagree’) to 6 (‘strongly agree’), with higher scores indicating greater age-friendliness.

The reliability (Cronbach’s alpha) estimates were 0.81 for Outdoor Spaces and Buildings (e.g., sufficient, safe and well-maintained green spaces and outdoor seating), 0.90 for Transportation (e.g., public transportation costs are affordable), 0.67 for Housing (e.g., Housing in safe areas, close to services and community), 0.88 for Social Participation (e.g., variety of activities offered to appeal to a diverse population of older people), 0.83 for Respect and Social Inclusion (e.g., older people are recognized by the community for their past as well as their present contributions), 0.82 for Civic Participation and Employment (e.g., promotion of flexible paid opportunities for older people to work), 0.83 for Communication and Information (e.g., an effective communication system reaches community residents of all ages), and 0.83 for Community Support and Health Services (e.g., health services are easily accessible by all means of transport).

The original questionnaire developed by [6] contains also an eight-item scale to assess Sense of Community along with dimensions of needs, fulfilment, group membership, influence, and emotional connection [36]. We have opted for a simplified four-item scale that uses the McMillan and Chavis model [37]. Sense of community was defined as ‘‘a feeling that members have of belonging, a feeling that members matter to one another and to the group, and a shared faith that members’ needs will be met through their commitment to be together’’ [37].

The sense of community was rated on a five-point Likert-type scale ranging from “strongly disagree” to “strongly agree” to describe a participant’s community experiences. A high score on the SOC indicated a better sense of community. Cronbach’s Alpha for Sense of Community is 0.84.

### 2.5. Statistical Analysis

Descriptive statistics were calculated for the overall sample for the eight domains of AFC scale, as well as for sense of community, to investigate the perception of the elderly with respect of the age-friendliness of the city of Macerata. Domain scores were estimated by the average of the scores of the individual items under the corresponding domains. Domain scores were calculated only if over half of the aspects under that domain had valid responses.

Furthermore, the association of socio-demographic predictors with Sense of Community was tested to understand the relationship between the two. Multiple regression was carried out. Similarly with respect to the AFC domains.

## 3. Results

A sample of 163 older adults was collected, but 24 participants were removed because they had none or only one out of four values in the sense of community subscale. With these removed, every remaining participant had at least 50% of data available, resulting in 139 questionnaires analyzed. Demographic information for all the participants is provided in Table 1.

The mean age of the overall sample is 65.25 ranging from 50 to 90. There are 63 men (45.3%) and 76 women (54.7%).

Most of the subjects are married (58.27%), own their own house (77.7%) live with family (72.66%), and have education at secondary level and above (87.7%). The average length of residence in neighborhood is 28.02 years. Slightly more than half are retirees (51.8%). The majority has prior experience delivering care to elderly (70.5%) and to children (64.75%). Elderly community centers or services are used by a minority (29.5%). In terms of self-reported health, the majority (59.7%) rates overall health as good, very good, or excellent. The majority rates income as (very) sufficient (57.6%), while 20.14% rates it just enough.

Table 2 shows the mean scores of AFC items and domains. They vary across domains: housing and health services rank the highest, that is 3.79 and 3.34, respectively. On the other hand, outdoor spaces and buildings (2.89), as well as civic participation and employment (2.81), rank the lowest. The other domains rank in between. Sense of Community ranks second highest, that is 3.41.

Table 3 shows that the mean itemized scores varied from accessibility of interior spaces of houses (highest rated item: 4.16) to maintenance and accessibility of sidewalks and arrangements of special customer services for elderly (lowest rated items: 2.16). In general, several items in the outdoor spaces and buildings domain received rather low scores, including reporting lack of public toilets (2.17), low maintenance and safety of green areas (2.84), and little attention for pedestrian crossing streets (2.80). The only positive score in this domain is with respect to availability of accessible commercial services (i.e., banks, post offices, supermarkets) in one’s neighborhood.

Similar negative perception is attested with respect to transportation in which 7 of the 12 items score below 3.00. Citizens are skeptical about the reliability of public transport (2.30); they highlight the low availability of taxis (2.97) and lack of alternative transport for less accessible areas (2.73) and the bad conditions of roads (2.67). The best score is reserved for the attentive behavior of drivers (3.55).

With respect to communication and information, citizens are aware that internet and social media use could be beneficial for elderly since they facilitate social inclusion (4.00), but they acknowledge lack of knowledge on their use (2.49), as well as lack of available courses (2.67). More information with respect to these items can be found in [38].

Community Support and Health services is a domain that scores rather high, as already mentioned, since 5 of the 7 items score above 3.00. Citizens are especially positive with respect to the accessibility of health services (3.53) and the manner of their personnel (3.60). Similarly, a score of 3.71 is assigned to proximity between old age’s home and services, while home care services scores 3.37.

Citizens do feel part of the community understood as their neighborhood as well as family, friends, and associations; this is especially the case with respect to emotional connection that scores rather high (3.91), as well as group membership (3.45).

In order to gain more insights on Sense of Community, it was regressed on socio-demographic values. It was found that age, education, and self-rated health were associated with Sense of Community. More specifically, older age predicts a higher Sense of Community, similarly with respect to subjective health being rated as good, very good, and excellent. High education level predicts lower sense of community compared to lower education level (Table 4).

Furthermore, Sense of Community was regressed on the eight AFC domains. In this model, outdoor spaces and buildings, transportation, and health services are significant predictors for Sense of Community keeping every other variable equal. The mean score for the domain has been used (Table 5).

## 4. Discussion

This is the first attempt to assess quantitatively the age-friendliness of Macerata, a city located in rural surroundings within the Marche region, in Central Italy. The study has investigated the perceived age-friendliness of the city, among old adults and the elderly, based on the domains of the WHO AFC framework. Opinion has been sought by members of the community from a variety of ages ranging from 55 to 90 years.

This is an urban environment characterized by longevity, as the whole Marche region, which is becoming a preferred destination for international retirement migration [29]. One might wonder whether this might be the consequence of good infrastructure, services, and social inclusion. It is thus relevant to investigate the perception of elderly with respect to the age-friendliness of the city by also considering its peculiarity of being an urban context with strong rural background.

The findings show that the members of the community are not very satisfied with respect to the organization and maintenance of public spaces as well as of transportation, highlighting lack of good infrastructural services. Similarly with respect to social inclusion, which as domain does not score high since the elderly do not feel that they are particularly valued. Health and social services are appreciated in comparison to other services, in particular accessibility and manner of personnel are valued. Respondents are particularly satisfied about their own housing situation: houses are in safe areas, are close to services and to the rest of the community, and can be easily adapted to changing needs of the ageing population. A high percentage of the analyzed sample owns a house and has lived for an average of 28 years in the same place, which is quite a long period.

The analysis of the AFC domains shows that the city is not particularly age-friendly since infrastructure and services are generally below average except for health and social services. However, the elderly are quite satisfied with their own housing and with the community they live in given that sense of community is the second highest score.

One might wonder whether the AFC WHO domains that have been envisaged to assess age friendliness in urban contexts and have been analyzed mainly in the context of big, high-density, global cities might be appropriate and good indicators for cities with strong rural roots such as Macerata. The framework has been originally proposed using indicators that conceptualize metropolitan settings. It could be that the diverse needs of cities set in rural surroundings might not be adequately addressed within the framework [39].

Research on age-friendliness in rural and remote communities has been less prominent [40,41,42,43,44]. In particular, [42] lists a number of weaknesses related to ageing in rural settings such as inadequate infrastructure; geographical distances and isolation; limited availability of services, including social and health services (cf. also [45,46,47]) and lack of specialized expertise. On the positive side, strengths include strong social ties within the community since people know each other and take care of each other (cf. also [47,48]), strong sense of place and commitment to community issues, healthy lifestyle, and easy access to the natural environment.

It seems that Macerata, as a city set in a rural context, shares some of the strengths of rural environments described in [42] without sharing all the weaknesses. In particular, sense of community is attested, which is a feature that characterizes rural settings, understood as sense of place and commitment to the community with people taking care of others. The survey reveals that care activities are prominent in Macerata, as in the case of older adults that take care of elderly parents, as well as grandparents taking care of their grandchildren: findings show high percentage of commitment in this respect.

This is probably due to a strong sense of family, that is typical of the Mediterranean culture. As argued in [31], Italy is characterized by a strong familistic welfare model that relies on families for intergenerational care responsibilities with minimal policies and financial support from the state. Most of the respondents in this study are married and live in a family context. The Italian welfare system is thus strongly based on extended family solidarity and responsibility for care, including care for older people [49,50]. As already mentioned, 70% of the respondents have experience taking care of older people aged above 65. However, in Italy, care activities mainly rely on women [31], as also attested in this survey in which the percentage of women taking care of elderly is 76% against 65% of men respondents. On the other hand, often reciprocity is attested since elderly are not only taken care of, but they also take care of their grandchildren (i.e., 64.75%).

It should be noticed that care ethics acknowledges a double dimension in caring activities, such as the need for care and need to care [51,52,53] that is also attested by elderly. Caring activities might play a role in determining an appropriate setting for ageing gracefully in Macerata [53,54].

Caring activities play a crucial role also in Japan and are encouraged through policies that are being developed to give the possibility to retired people to carry out activities that involve agriculture, food, and community support [55]. It can be shown that volunteering, especially if it involves nature, may have health benefits for the elderly since it triggers physical activity and thus promote health and help reducing expenditure for health care systems [56,57]. In this respect, volunteering and civic participation should be more encouraged in Macerata and respondents highlight lack of opportunities. There is the possibility for improvement through policies, projects, and employment opportunities, especially in the context of agriculture, given the rural setting in which Macerata is located.

An investigation of the socio-demographic features shows that older people who are low-educated and with good health seem the most satisfied with their community. These findings show similarity with those of [58] that investigated rural communities in which older people scored higher with respect to sense of community. This might be due to the fact that age might lead to higher interaction and more relationships.

The relatively high score given by elderly to health services and community support shows that in this domain Macerata does not exhibit the weakness found in the case of rural environments. However, it shares with rural environments the limited infrastructure especially with respect to transportation, which can create problems for the mobility of old adults [59]. While this weakness can be problematic in countries where lack of transportation might isolate individuals and communities due to big distances, the situation is different in the context of Macerata since geographical distances are not very big with services being located within walking distance, as also acknowledged by respondents.

The eight WHO domains do not address in detail the role played by technology in the age-friendliness of cities, as also acknowledged by [60] that highlights the importance of technology as integral part of age-friendliness. Additional questions were therefore added in this study, and the findings show a low uptake of technology among the respondents (cf. also [38]). In the study, [61] shows that urban respondents have more affinity with technology than rural ones, and in the case of the respondents of our survey, they seem to pattern more like rural respondents.

Technology could be a solution to urban ageing since it can be employed for health monitoring, automated assessment of the need for assistance, and more generally for smart homes [62]. On the other hand, while technology in the form of internet and social media might improve social inclusion, the low uptake exhibited by the respondents might have a positive side-effect and stimulate a stronger connection to the environment and to the community. In the case of Macerata, however, the environment is not represented by the public urban spaces addressed in the questionnaires to which rather low scores were assigned but the beautiful rural setting that characterizes the Marche region, which creates the basis for an age-friendly context.

It could be that other factors not considered within the eight AFC WHO domains might thus play a role in determining the favorable context for ageing that drives longevity in Macerata, such as the beautiful rural setting in which the city is located. Place can have an important impact on the quality of life of older people and to successful active ageing [63]. In fact, the connection between landscape, soil, and food is what determines the good quality of life of the Marche region and what attracts the elderly from Northern Europe to settle in its medieval cities of which Macerata is a typical example [29].

The present study shows some limitations: the subjects were recruited among the personnel of administrative offices while older people were recruited in old people centers, churches, and community halls, which may be a source of selection bias by omitting those who were home-bound and have limited access to outdoor environments. Furthermore, the data were collected before COVID-19 that had an impact on the life and the well-being of the community.

## 5. Conclusions

Empirical research with focus on the age-friendliness of Italian cities is very limited despite the rapidly growing elderly population. The study presented in this paper aims to fill this gap by focusing on the city of Macerata, as representative case of an urban community in the Marche Region, Italy.

The paper has investigated the perception of the elderly residents on age-friendliness by means of a quantitative analysis based on a questionnaire that relies on the well-established eight WHO AFC domains. In addition, sense of community has been assessed and how the older residents relate to it.

Most research on age-friendliness has focused on urban settings (i.e., North America, Asia Pacific Region) or rural ones (i.e., Canada and Australia) but has not considered contexts that share features of both. The case study analyzed in this paper is rather innovative in this respect since it presents an environment that combines urban and rural characteristics. Findings reveal that the elderly respondents are not generally satisfied about the services being offered and the infrastructure of the city but appreciate health services and community support; furthermore, respondents exhibit strong sense of community. It seems thus that Macerata patterns like rural settings in showing strong social ties, as well as sense of place and commitment to the community, but shares with urban settings, good health services and community support. In future research, it would be relevant to assess whether sense of community could be a predictor for life satisfaction and well-being [64].

At the policy level, improvements are necessary with respect to the physical infrastructure, especially transportation as well as outdoor spaces. This is similar with respect to services and activities that foster social inclusion and participation. The strong sense of community exhibited by the respondents in this study highlights the attachment to their environment, which could be exploited by policy makers. It would be desirable to involve old adults and elderly as actors in setting the agenda for age-friendly developments.

In particular, the survey based on the eight WHO domains highlights the urban features exhibited by Macerata in determining its age-friendliness but does not consider the rural aspects that might also play a role. Similarly, the strong family culture typical of Mediterranean countries is not accounted for by the domains investigated that focus more on a community driven sociality instead of a family-oriented one. Those are aspects that should be considered in future research.

It might be the combination of rural and urban characteristics typical of many Italian cities that contributes to longevity and to strong sense of community making up for poor infrastructure and average services, which nevertheless need improvement. It is this combination that makes the Marche region and its cities a new frontier for international retirement migration.

## Figures and Tables

**Table 1:** Descriptive statistics.

		Years	sd
Age mean		65.25	10.71
Length of residence in neighborhood		28.02	16.81
		*n* (total = 139)	%
Sex	Male	63	45.32
	Female	76	54.68
Marital Status	Currently not married	58	41.73
	Currently married	81	58.27
Education level	Primary and below	17	12.23
	Secondary	78	56.12
	Post-secondary	44	31.65
Type of housing	Rental	26	18.71
	Private permanent	108	77.7
	Other	5	3.6
Living arrangement	Living alone	33	23.74
	Not living alone	101	72.66
	Other	5	3.6
Economic activity status	Employed	59	42.45
	Unemployed	3	2.16
	Retired	72	51.80
	Other	5	3.6
Self-rated health	Poor/Fair	55	39.57
	Good/Very good/excellent	83	59.71
Prior experience of delivering care to elderly	Yes	98	70.05
	No	40	28.78
Prior experience of delivering care to children	Yes	93	64.75
	No	46	33.09
Use of elderly service centers	Yes	41	29.5
	No	95	68.35
Personal disposable income	(Very) sufficient	80	57.55
	Just enough	28	20.14
	(Very) insufficient	31	22.30

**Table 2:** Age friendly city domains in decreasing order based on mean score.

AFC Domains	Mean	SD
Housing	3.79	0.89
Sense of community	3.41	0.99
Health services	3.34	0.92
Social Participation	3.23	1.04
Transportation	3.07	0.99
Communication and information	3.05	0.87
Respect and social inclusion	3.05	0.97
Outdoor spaces and buildings	2.89	0.81
Civic participation and employment	2.81	1.2

**Table 3:** Mean scores of the WHO AFC items.

Item	Mean	SD	Median	Min	Max
A1 Accessible and safe locations housing	4.15	1.07	4.00	2.00	6.00
A2 Interior Spaces and Level Surfaces of Housing	4.16	1.21	4.00	1.00	6.00
A3 Home Modification Options and Supplies	3.79	1.36	4.00	1.00	6.00
A4 Housing for Frail and Disabled Elders	2.81	1.24	3.00	1.00	6.00
*Domain 1: Housing*					
CI1 Effective Communication System	3.29	1.23	3.00	1.00	6.00
CI2 Information and Broadcasts of Interest to Elderly	2.93	1.17	3.00	1.00	6.00
CI3 Information to Isolated Individuals	2.57	1.14	2.00	1.00	5.00
CI4 Printed information	3.12	1.43	3.00	1.00	6.00
CI5 Automated Telephone Answering Services	2.58	1.27	3.00	1.00	5.00
CI6 Electronic Devices and Equipment	3.28	1.49	3.00	1.00	6.00
CI7 Access to Computers and Internet	3.00	1.41	3.00	1.00	6.00
CI8 Internet courses to elderly	2.67	1.34	2.00	1.00	6.00
CI9 Social media use	2.49	1.37	2.00	1.00	6.00
CI10 Internet and social media for social inclusion	4.00	1.51	4.00	1.00	6.00
*Domain 2: Communication and Information*					
B1 Cleanliness	3.10	1.31	3.00	1.00	6.00
B2 Adequacy, Maintenance and Safety	2.84	1.35	3.00	1.00	6.00
B3 Sidewalks maintenance and accessibility	2.16	1.22	2.00	1.00	6.00
B4 Drivers’ Attitude at Pedestrian Crossings	2.80	1.23	3.00	1.00	5.00
B5 Outdoor Lighting and Safety	3.69	1.50	4.00	1.00	6.00
B6 Accessibility of Commercial Services	4.04	1.36	4.00	1.00	6.00
B7 Arrangement of Special Customer Service for elderly	2.16	1.17	2.00	1.00	6.00
B8 Building Facilities	2.98	1.31	3.00	1.00	6.00
B9 Public Washrooms	2.17	1.11	2.00	1.00	6.00
*Domain 3: Outdoor Spaces and Buildings*					
C1 Affordability of Public Transport	2.92	1.41	3.00	1.00	6.00
C2 Reliability of Public Transport	2.30	1.32	2.00	1.00	6.00
C3 Coverage of Public Transport Network	3.07	1.49	3.00	1.00	6.00
C4 Condition of Public Transport Vehicles	3.54	1.38	4.00	1.00	6.00
C5 Specialized Transportation for disabled people	2.93	1.46	3.00	1.00	6.00
C6 Behaviour of Public Transport Drivers	3.55	1.45	4.00	1.00	6.00
C7 Transport Stops and Stations	3.52	1.33	4.00	1.00	6.00
C8 Public Transport Information	3.14	1.41	3.00	1.00	6.00
C9 Alternative Transport in Less Accessible Areas	2.73	1.61	2.00	1.00	6.00
C10 Taxi	2.93	1.33	3.00	1.00	6.00
C11 Roads	2.67	1.27	3.00	1.00	6.00
C12 Reserved parking for elderly	2.99	1.38	3.00	1.00	6.00
*Domain 4: Transportation*					
E1 Mode of Participation	3.54	1.38	4.00	1.00	6.00
E2 Adequate event time	3.12	1.30	3.00	1.00	6.00
E3 Participation Costs	3.50	1.30	4.00	1.00	6.00
E4 Information about Activities and Events	3.22	1.34	3.00	1.00	6.00
E5 Variety of Activities	2.87	1.29	3.00	1.00	6.00
E6 Variety of Venues for Elders’ Gatherings	3.18	1.24	3.00	1.00	6.00
E7 Outreach Services to People at Risk of Social Isolation	2.75	1.19	3.00	1.00	6.00
*Domain 5: Social Participation*					
F1 Consultation from Different Services	2.64	1.29	3.00	1.00	6.00
F2 Variety of Services and Goods	3.28	1.29	3.00	1.00	6.00
F3 Manner of Service Staff	3.85	1.14	4.00	1.00	6.00
F4 Visibility and Media Depiction	3.14	1.23	3.00	1.00	6.00
F5 School as Platform for Intergeneration Exchange	2.57	1.31	2.00	1.00	6.00
F6 Social Recognition	2.85	1.37	3.00	1.00	6.00
F7 Availability of services for elderly without means	2.73	1.27	3.00	1.00	6.00
*Domain 6: Respect and Social Inclusion*					
H1 Options for Older Volunteers	3.13	1.28	3.00	1.00	6.00
H2 Paid Work Opportunities for Older People	2.46	1.19	2.00	1.00	6.00
H3 Promote Qualities of Older Employees	2.61	1.33	2.00	1.00	6.00
*Domain 7: Civic Participation and Employment*					
L1 Adequacy of Health and Community Support Services	3.17	1.28	3.00	1.00	6.00
L2 Home Care Services	3.37	1.29	3.00	1.00	6.00
L3 Accessibility social and health services	3.53	1.36	3.00	1.00	6.00
L4 Proximity between Old Age Homes and Services	3.71	1.28	4.00	1.00	6.00
L5 Manner of health service staff	3.60	1.21	4.00	1.00	6.00
L6 Economic barriers to Health and Community Support Services	2.98	1.21	3.00	1.00	6.00
L7 Community Emergency Planning	2.67	1.23	3.00	1.00	6.00
*Domain 8: Community Support and Health Services*					
AC1 needs fulfillment	3.15	1.20	3.00	1.00	5.00
AC2 group membership	3.45	1.22	4.00	1.00	5.00
AC3 influence	3.17	1.29	3.00	1.00	5.00
AC4 emotional connection	3.91	1.04	4.00	1.00	5.00
*Sense of Community*					

**Table 4:** Multiple regression results for sense of community with socio-demographic variables.

	Beta	Std. Error	t-Value	*p*
Sex	0.22	0.17	1.3	0.193
Age	0.039	0.01	2.85	0.005 **
Marital Status—currently Married vs. not married	−0.23	0.21	−1.08	0.282
Educational level—Secondary vs. primary	−0.39	0.25	−1.54	0.125
Educational level—Post-secondary vs. primary	−0.87	0.31	−2.8	0.006 **
Type of housing—Private permanent vs. rental	0.11	0.22	0.48	0.634
Type of housing—Other vs. rental	0.37	0.47	0.78	0.436
Length of residence in neighborhood	0	0.01	−1.7	0.092
Living arrangement—Not living alone vs. other	0.67	0.49	1.36	0.176
Living arrangement—Living alone vs. other	0.44	0.52	0.84	0.4
Economic activity status—Unemployed vs. employed	−0.04	0.67	−0.06	0.95
Economic activity status—Retired vs. employed	−0.23	0.28	−0.81	0.418
Economic activity status Other—vs. employed	−0.19	0.48	−0.4	0.689
Self-rated health—Good/very good/excellent vs. poor	0.56	0.17	3.37	0.001 **
R2	0.26			

Significance level ** *p* < 0.01.

**Table 5:** Multiple regression for sense of community on AFC domains.

	Beta	Std. Error	t-Value	*p*
Civic participation and employment	0.11	0.08	1.29	0.199
Housing	−0.01	0.12	−0.09	0.929
Communication and Information	0.12	0.13	0.88	0.380
Outdoor spaces and buildings	−0.34	0.15	−2.26	0.026 *
Transportation	0.34	0.13	2.51	0.014 *
Social participation	0.20	0.12	1.71	0.090
Respect and Social Inclusion	−0.17	0.14	−1.24	0.216
Health services	0.44	0.14	3.15	0.002 **
R2	0.47			

Significance level * *p* < 0.05; ** *p* < 0.01.

## Data Availability

Not applicable.

## References

[1] World Health Organization (2002). Active Ageing: A Policy Framework.

[2] Lui C.W., Everingham J.A., Warburton J., Cuthill M., Bartlett H. (2009). What makes a community age-friendly: A review of international literature. Australas. J. Ageing.

[3] (2023). World Health Organization. https://www.who.int/teams/social-determinants-of-health/demographic-change-and-healthy-ageing/age-friendly-environments.

[4] World Health Organization (2007). Checklist of Essential Features of Age-Friendly Cities.

[5] World Health Organization (2007). Global Age-Friendly Cities: A Guide.

[6] Wong M., Chau P.H., Cheung F., Phillips D.R., Woo J. (2015). Comparing the age-friendliness of different neighbourhoods using district surveys: An example from Hong Kong. PLoS ONE.

[7] Wong M., Yu R., Woo J. (2017). Effects of Perceived Neighbourhood Environments on Self-Rated Health among Community-Dwelling Older Chinese. Int. J. Environ. Res. Public Health.

[8] Au A., Lai D.W.L., Yip H.M., Chan S., Lai S., Chaudhury H., Scharlach A., Leeson G. (2020). Sense of Community Mediating Between Age-Friendly Characteristics and Life Satisfaction of Community-Dwelling Older Adults. Front. Psychol..

[9] Sun Y., David R., Phillips M.W. (2018). A study of housing typology and perceived age-friendliness in an established Hong Kong new town: A person-environment perspective. Geoforum.

[10] Yu R., Moses W., Jean W. (2018). Perceptions of neighborhood environment, sense of community, and self-rated health: An age-friendly city project in Hong Kong. J. Urban Health.

[11] Kitreerawutiwong N., Keeratisiroj O., Mekrungrongwong S. (2020). Predictive Factors for the Sense of Community Belonging Among Older Adults in Lower Northern Thailand. Iran. J. Psychiatry Behav. Sci..

[12] Dikken J., van den Hoven R.F.M., van Staalduinen W.H., Hulsebosch-Janssen L.M.T., van Hoof J. (2020). How Older People Experience the Age-Friendliness of Their City: Development of the Age-Friendly Cities and Communities Questionnaire. Int. J. Environ. Res. Public Health.

[13] Plouffe L., Kalache A., Voelcker I., Moulaert T., Garon S. (2016). A critical review of the WHO age-friendly cities methodology and its implementation. Age-Friendly Cities and Communities in International Comparison: Political Lessons, Scientific Avenues, and Democratic Issues.

[14] Torku A., Chan A.P.C., Yung E.H.K. (2020). Age-friendly cities and communities: A review and future directions. Ageing Soc..

[15] Finkelstein R., Garcia A., Netherland J., Walker J. (2008). Toward an Age-Friendly New York City: A Findings Report.

[16] Biggs S., Tinker A. (2007). What Makes a City Age-Friendly? London’s Contribution to the World Health Organisation’s Age-Friendly Cities Project.

[17] Plouffe L., Kalache A. (2010). Towards global age-friendly cities: Determining urban features that promote active aging. J. Urban Health.

[18] Buffel T., McGarry P., Phillipson C., De Donder L., Dury S., De Witte N., Smetcoren A., Verté D. (2014). Developing Age-Friendly Cities: Case Studies From Brussels and Manchester and Implications for Policy and Practice. J. Aging Soc. Policy.

[19] Rémillard-Boilard S., Buffel T., Phillipson C. (2021). Developing Age-Friendly Cities and Communities: Eleven Case Studies from around the World. Int. J. Environ. Res. Public Health.

[20] Ruza J., Kim J.I., Leung I., Kam C., Ng S.Y.M. (2015). Sustainable, age-friendly cities: An evaluation framework and case study application on Palo Alto, California. Sustain. Cities Soc..

[21] Dandy K., Bollman R.D. (2008). Seniors in rural Canada. Rural and Small Town Canada Analysis Bulletin.

[22] Federal, Provincial, Territorial Ministers Responsible for Seniors (2007). Age-Friendly Rural and Remote Communities: A Guide.

[23] Winterton R. (2016). Organizational responsibility for age-friendly social participation: Views of Australian rural community stakeholders. J. Aging Soc. Policy.

[24] Neville S., Napier S., Adams J., Wham C., Jackson D. (2016). An integrative review of the factors related to building age-friendly rural communities. J. Clin. Nurs..

[25] Walsh K., O’Shea E., Scharf T., Murray M. (2012). Ageing in changing community contexts: Cross-border perspectives from Ireland and Northern Ireland. J. Rural Stud..

[26] United Nations, Department of Economic and Social Affairs, Population Division (2019). World Population Ageing 2019: Highlights (ST/ESA/SER.A/430).

[27] Clavis D.M., Wandersman A. (1990). Sense of Community in the Urban-Environment—A catalyst for participation and community-development. Am. J. Community Psychol..

[28] Talo C., Mannarini T., Rochira A. (2014). Sense of community and community participation: A meta-analytic review. Soc. Indic. Res..

[29] King R., Cela E., Fokkema T., Morettini G. (2021). International retirement and later- life migrants in the Marche Region. Italy: Materialities of landscape, ‘home’, lifestyle and consumption. Ageing Soc..

[30] ISTAT (2018). Il Futuro Demografico del Paese. Previsioni Regionali Della Popolazione Residente al 2065 (Base 1.1.2017). Report. https://www.istat.it/it/files/2018/05/previsioni_demografiche.pdf.

[31] Zannella M., Principi A., Lucantoni D., Barbabella F., Di Rosa M., Domínguez-Rodríguez A., Socci M. (2021). Active Ageing: The Need to Address Sub-National Diversity. An Evidence-Based Approach for Italy. Int. J. Environ. Res. Public Health.

[32] Cela E., Moretti E. (2019). Popolazione e Invecchiamento Nelle Marche.

[33] Hira K.H., Mugenda M.O. (1998). Predictors Of financial satisfaction: Differences between retirees and non-retirees. Finan. Consel. Plann. Educ..

[34] Garrett S., James R.N. (2013). Financial ratios and perceived household financial satisfaction. J. Finan. Ther..

[35] World Health Organization (2015). Measuring Age-Friendliness of Cities: A Guide to Using Core Indicators.

[36] Peterson N.A., Speer P.W., McMillan D.W. (2008). Validation of a brief sense of community scale: Confirmation of the principal theory of sense of community. J. Commun. Psychol..

[37] McMillan D.W., Chavis D.M. (1986). Sense of community: A definition and theory. J. Commun. Psychol..

[38] Monachesi P. (2019). Sustainable Development and ICT Use Among Elderly: A Comparison Between the Netherlands and Italy. Human Aspects of IT for the Aged Population, Design for the Elderly and Technology Acceptance.

[39] Montayre J., Foster J., Zhao I.Y., Kong A., Leung A.Y.M., Molassiotis A., Officer A., Mikton C., Neville S. (2022). Age-friendly interventions in rural and remote areas: A scoping review. Australas J Ageing..

[40] Spina J., Menec V.H. (2015). What community characteristics help or hinder rural communities in becoming age-friendly? Perspectives from a Canadian prairie province. J. Appl. Gerontol..

[41] Menec V.H., Hutton L., Newall N., Nowicki S., Spina J., Veselyuk D. (2015). How ‘age-friendly’ are rural communities and what community characteristics are related to age-friendliness? The case of rural Manitoba, Canada. Ageing Soc..

[42] Menec V., Bell SNovek S., Minnigaleeva G.A., Morales E., Titus Ouma P.P.M.M.M.S., Parodi J.F., Winterton R. (2015). Making Rural and Remote Communities More Age-Friendly: Experts’ Perspectives on Issues, Challenges, and Priorities. J. Aging Soc. Policy.

[43] Buffel T., Phillipson C. (2018). A manifesto for the age-friendly movement: Developing a new urban agenda. J. Aging Soc. Policy.

[44] Russell E., Skinner M., Colibaba A. (2021). Developing rural insights for building age-friendly communities. J. Rural Stud..

[45] Hanlon N., Halseth G. (2005). The greying of resource communities in northern British Columbia: Implications for health care delivery in already-underserviced communities. Can. Geogr..

[46] Winterton R., Warburton J. (2011). Does place matter? Reviewing the experience of disadvantage for older people in rural Australia. Rural Soc..

[47] Winterton R., Warburton J. (2011). Models of care for socially isolated older rural carers: Barriers and implications. Rural Remote Health.

[48] Norstrand J.A., Xu Q. (2012). Social capital and health outcomes among older adults in China: The urban–rural dimension. Gerontol..

[49] Ferrera M. (1996). The “southern model” of social welfare in Europe. J. Eur. Soc. Policy.

[50] Saraceno C. (2016). Varieties of familialism: Comparing four southern European and East Asian welfare regimes. J. Eur. Soc. Policy.

[51] Lloyd L. (2004). Mortality and morality: Ageing and the ethics of care. Ageing Soc..

[52] Kittay E. (2011). The Ethics of Care, Dependence, and Disability. Ratio Juris.

[53] Carella V., Monachesi P. (2018). Greener through Grey? Boosting Sustainable Development through a Philosophical and Social Media Analysis of Ageing. Sustainability.

[54] Monachesi P. (2021). Audiopapers Mediating Sustainable Cities. Elderly Care and Environmental Sustainability. Audio Paper 3: Mediating Sustainable Cities: Elderly Care and Environmental Sustainability. https://mediatingsustcities.sites.uu.nl/audio-paper-3-mediating-sustainable-cities-elderly-care-and-environmental-sustainability/.

[55] Muramatsu N., Akiyama H. (2011). Japan: Super-Aging Society Preparing for the Future. Gerontol..

[56] Kaplan S. (1995). The restorative benefits of nature. Toward an integrative framework. J. Environ. Psychol..

[57] Ulrich R., Simons R., Losito B., Fiorito B., Miles M., Zelson M. (1991). Stress Recoveryduring Exposure to Natural and Urban Environments. J. Environ. Psychol..

[58] Wilkinson D. (2008). Individual and Community Factors Affecting Psychological Sense Of Community, Attraction, and Neighboring in Rural Communities. Can. Rev. Sociol. Rev. Can. De Sociol..

[59] Ryser L., Halseth G. (2012). Resolving mobility constraints impeding rural seniors’ access to regionalized services. J. Aging Soc. Policy.

[60] Marston H.R., van Hoof J. (2019). “Who doesn’t think about technology when designing urban environments for older people?” A case study approach to a proposed extension of the WHO’s age-friendly cities model. Int. J. Environ. Res. Public Health.

[61] Calvert J.F., Kaye J., Leahy M., Hexem K., Carlson N. (2009). Technology use by rural and urban oldest old. Technol. Health Care.

[62] van Hoof J., Kazak J.K., Perek-Białas J.M., Peek S.T.M. (2018). The challenges of urban ageing: Making cities age-friendly in Europe. Int. J. Environ. Res. Public Health.

[63] Andrews G.J., Phillips D.R. (2005). . Ageing and Place: Perspectives, Policy, Practice.

[64] Buckley T.D. (2022). A Scoping Review of Psychological Sense of Community among Community-Dwelling Older Adults. Int. J. Environ. Res. Public Health.

